# Opportunistic Community Screening of Chronic Chagas Disease Using a Rapid Diagnosis Test in Pharmacies in Barcelona (Catalonia, Spain): Study Protocol and Pilot Phase Results

**DOI:** 10.3389/ijph.2022.1605386

**Published:** 2022-11-30

**Authors:** Aroa Silgado, Pau Bosch-Nicolau, Adrián Sánchez-Montalvá, Ariadna Cervià, Jordi Gomez-i-Prat, Guillermo Bagaria, Cristina Rodriguez, Lidia Goterris, Núria Serre-Delcor, Inés Oliveira-Souto, Fernando Salvador, Israel Molina, Elena Sulleiro

**Affiliations:** ^1^ Department of Microbiology, Vall d’Hebron University Hospital, Universitat Autònoma de Barcelona, PROSICS Barcelona, Barcelona, Spain; ^2^ Department of Infectious Diseases, Vall d’Hebron University Hospital, Universitat Autònoma de Barcelona, PROSICS Barcelona, Barcelona, Spain; ^3^ Centro de Investigación Biomédica en Red de Enfermedades Infecciosas, Instituto de Salud Carlos III, Madrid, Spain; ^4^ Official College of Pharmacists of Barcelona, Barcelona, Spain

**Keywords:** Chagas disease, opportunistic screening, community pharmacies, rapid test, dried blood

## Abstract

**Objectives:** This study aimed to report the protocol and results from the pilot phase of an opportunistic CP-based CD screening program in Barcelona, Spain.

**Methods:** Three strategies according to recruitment approach were designed: passive, active and active-community. The study process consisted of signing the informed consent form, recording the patient’s data in a web-based database system, and performing the rapid test and blood collection on dry paper.

**Results:** Nineteen pharmacies participated and 64 patients were included during the pilot phase of the study. The rapid diagnostic test (RDT) was positive in 2/64 (3.13%) cases. Of the 49 DBS samples that arrived at the laboratory, 22 (45%) were collected incorrectly. After quantitative and qualitative assessment of the program, the dry paper sample and passive strategy were ruled out.

**Conclusion:** DBS sampling and the passive strategy are not suitable for CD screening in community pharmacies. There is a need to expand the number of participating pharmacies and individuals to determine whether conducting a RDT in community pharmacies is an effective screening method to increase access to CD diagnosis in a non-endemic area.

## Introduction

Chagas disease (CD), a life-threatening disease caused by the protozoan *Trypanosoma cruzi*, affects between 6 and 7 million people worldwide (1). It is estimated that nearly 10,000 deaths per year are directly caused by CD, with an estimated global cost of 8 billion dollars per year in lost productivity and health costs (2). Although endemic in Latin America, the number of CD cases reported in non-endemic countries has increased due to population movements (3). In Europe, Spain has the highest number of people infected by *T. cruzi*, with an estimated 52,000 cases; however, approximately 70% remain undiagnosed (4).

In the absence of the vector, CD can be transmitted *via* vertical transmission, blood transfusion and organ transplantation (5). Accordingly, health authorities in Spain have implemented different measures to control CD transmission: screening in blood banks is mandatory (Royal Decree 1088/2005) and some Spanish regions established CD screening protocols in pregnant women from Latin America, such as Valencia (in 2008) (6), Galicia (in 2008) (7) and Catalonia (in 2015) (8). However, no screening program to control CD in the general Latin American population is implemented in Spain and CD diagnosis relies on the general physician’s decision. Unfortunately, time constraints and the healthcare workers’ poor knowledge of CD, inefficiencies in the diagnosis and referral process flows, together with barriers to health system access and stigmatization, limit the current strategy to diagnose people with CD (9). To overcome these barriers, community-based activities for screening and case detection allow us to identify at-risk individuals and thus limit potential associated complications through early detection and appropriate treatment (10).

The vast majority of CD patients living in non-endemic areas are in the chronic stage of the disease (11). Diagnosis of this phase is by means of serological tests (12); for mass-screening surveys, a rapid, sensitive and easy to perform and interpret point of care diagnostic test would be valuable (13). Rapid diagnostic tests (RDTs) to detect antibodies against *T. cruzi* have been developed (14). Additionally, Dried Blood Spots (DBS) from finger-prick blood can be safely collected, stored at room temperature and sent under conventional courier conditions (15).

Community pharmacies (CPs) are a private healthcare resource that work in an integrated and coordinated manner in the management of health processes of the population. CPs are valuable assets for screening strategies due to territorial balance, consumer confidence, capillarity and social cohesion, professionalism and qualification, and their dense and interconnected network (16). Previous screening initiatives involving CPs have been carried out in Spain for other pathologies, such as opportunistic, under request, HIV screening programs and systematic colorectal cancer screening programs, with positive results (17, 18). To our knowledge, no CD screening program has been carried out in community pharmacies.

This study aimed to describe the protocol and results from the pilot phase of an opportunistic CD screening program at CPs in Barcelona (Catalonia, Spain).

## Methods

### Stakeholders, Target Population and Study Design

This was an opportunistic CD screening intervention performed in CPs for people aged 18 or over, who are originally from one of the 21 endemic countries for CD, have been in an endemic area for more than 1 month or have a mother originally from an endemic country, even if the participant was born here. This project was financed by the Fundació Marató TV3 (project number 20182610) and supported by different referent organizations: College of Pharmacists of Barcelona, Vall d’Hebron University Hospital (HUVH), International Health Program of the Catalan Health Institute and XarChagas program. The pilot phase was performed from November 2019 to May 2021, at which time the strengths, weaknesses, opportunities, and threats (SWOT) analysis and relevant modifications to the final study protocol were made.

**FIGURE 1:**
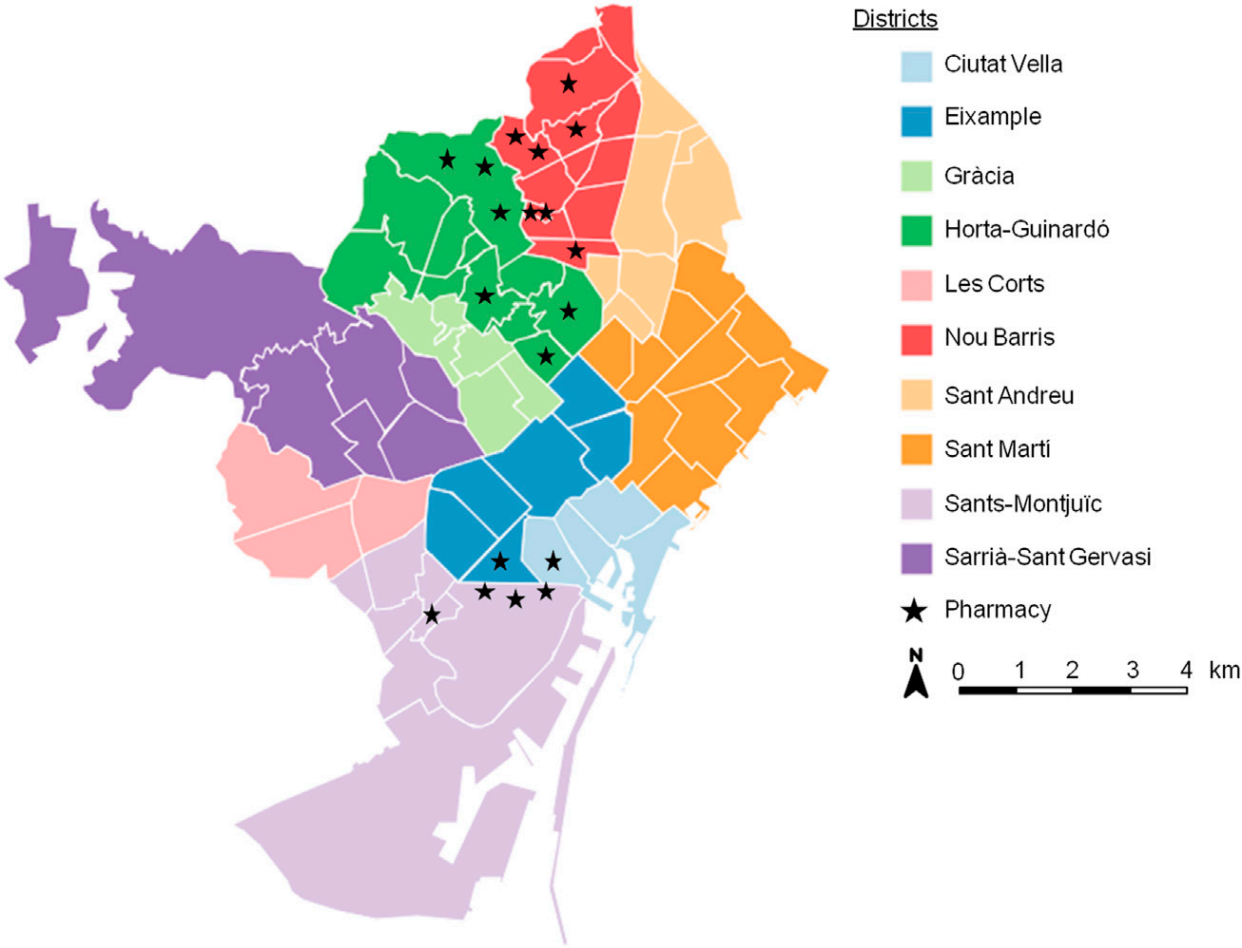
Distribution of the 19 pharmacies that participated in the pilot phase in the city of Barcelona. (Barcelona, Spain. 2019–2021). Modified from: https://www.instamaps.cat/

The screening study was carried out in three districts of Barcelona city (Figure 1) with a large population of Latin American migrants, according to the data of the last census in Barcelona (19): Nou Barris, Sants-Montjuïc and Horta-Guinardó. Participating pharmacies (N = 19) were selected based on different factors, including proactivity, experience in other screening programs (HIV and/or colorectal cancer), biological waste management training and certification, commitment to data protection procedures and facilities that ensure a comfortable and safe participant experience.

#### Opportunistic Screening Strategies

Three different strategies were used to approach the target population: 1) passive, 2) active and 3) active-community actions (Table 1). All participating CPs displayed posters offering CD screening at the entrance of the pharmacy, easily visible from the outside. In addition, informative leaflets were placed on the counter of the CP. Both posters and leaflets informed that the participant had the opportunity to take a free, rapid test to diagnose CD. In addition, the leaflets contained basic information about CD and reasons to be screened. Iconographic material was specially designed with the help of people affected with CD using a consensus decision-making process with the aim of drawing the attention of the Latin American population (Supplementary File S1).

**TABLE 1:** Characteristics of the three strategies presented in the CD screening program. (Barcelona, Spain. 2019–2021).

	Passive	Active	Active-community
Informative leaflets	**√** ^a^	**√**	**√**
Posters	**√**	**√**	**√**
Pharmacist offers rapid diagnosis tests	**x** ^b^	**√**	**√**
Involvement of community agents	**X**	**x**	**√**

^**a**^ Included in the strataegy.

^b^ Not included in the strategy.

i) Passive: implemented in Nou Barris, pharmacists had a wait-and-see attitude to recruit the target population, in this case the participant would request the RDT. ii) Active: implemented in Horta-Guinardó, pharmacists had a proactive attitude, where they explained and offered the RDT to Latin American consumers. iii) Active-community: implemented in those pharmacies located in the Sants-Montjuïc district. The iconographic material and the active attitude of the pharmacist were supplemented by the participation of community agents, who were tasked with conducting workshops/informative sessions in Latin American cultural associations and social meetings to encourage participation.

### Pharmacist Training

Pharmacists received training in CD and RDT performance by completing a 3 h course imparted by physicians, microbiologists and community agents. This course explained general aspects of CD (including epidemiology, diagnosis and treatment); how to use and interpret the RDT; how to collect DBS samples; the use of the information system tools; as well as how to approach the participant. In this sense, an “Expert Patient” (Latin American community worker) trained the pharmacist in techniques to identify and approach Latin American consumers (Supplementary File S2).

Participating pharmacies were invited to complete a brief anonymous survey with general questions about CD before and after the training. This survey consisted of 18 questions divided into four specific areas (epidemiology, transmission, clinical presentation and diagnosis/treatment). Moreover, another brief anonymous satisfaction survey was performed to evaluate the training course.

### Monitoring Plan

During the study, pharmacies received a personal visit from the training personnel (including a physician, a microbiologist, and a community health agent) to review the process, answer questions and correct any protocol deviations. To this end, a monitoring system was developed that included a guide for visiting and monitoring pharmacies (Standard Operational Procedure Monitoring Guideline). In addition to the on-site monitoring, pharmacies, lying in the last quartile according to the number of patients recruited, were telephoned and interviewed by an investigator regarding the hurdles hindering project implementation.

### Dissemination and Communication

Different collaborating entities were contacted to inform them of the implementation of the screening program, such as the health centers in the selected neighborhoods, the department of health of the Government of Catalonia, and different community organizations.

During the project, online stakeholder meetings were scheduled every 3 months or sooner under request from any stakeholder. A mailing list and a group in an instant message app were created for rapid communication.

### Information System Tools and Database

A web-based database platform was designed to record patient information, results obtained from the RDT and analysis of the DBS samples. This platform had personal authentication, search function, user privilege management and remote control.

The Official College of Pharmacists Information System centralized all information related to the processing and tracing of the RDT kits and DBS samples of the pharmacies and the microbiology laboratory of the HUVH.

### Study Procedures

#### Screening Methods

The pharmacist’s role, regardless of the strategy, was to check exclusion/inclusion criteria, inform about the importance of screening for CD, and explain the project’s objective, procedure, possible results and referral interventions. If the participant agreed, he/she signed two copies of the informed consent form; one copy is kept in the investigator file in the CP and the other is given to the participant. Later, the participant answered a short survey about sociodemographic characteristics and CD risk factors, relatives with the disease and previous testing history. Then, the RDT is performed using a blood drop from a finger-prick. All the procedures were free of charge for the patient. In addition to the rapid test, DBS samples were obtained from finger-prick blood on filter-paper cards.

**FIGURE 2:**
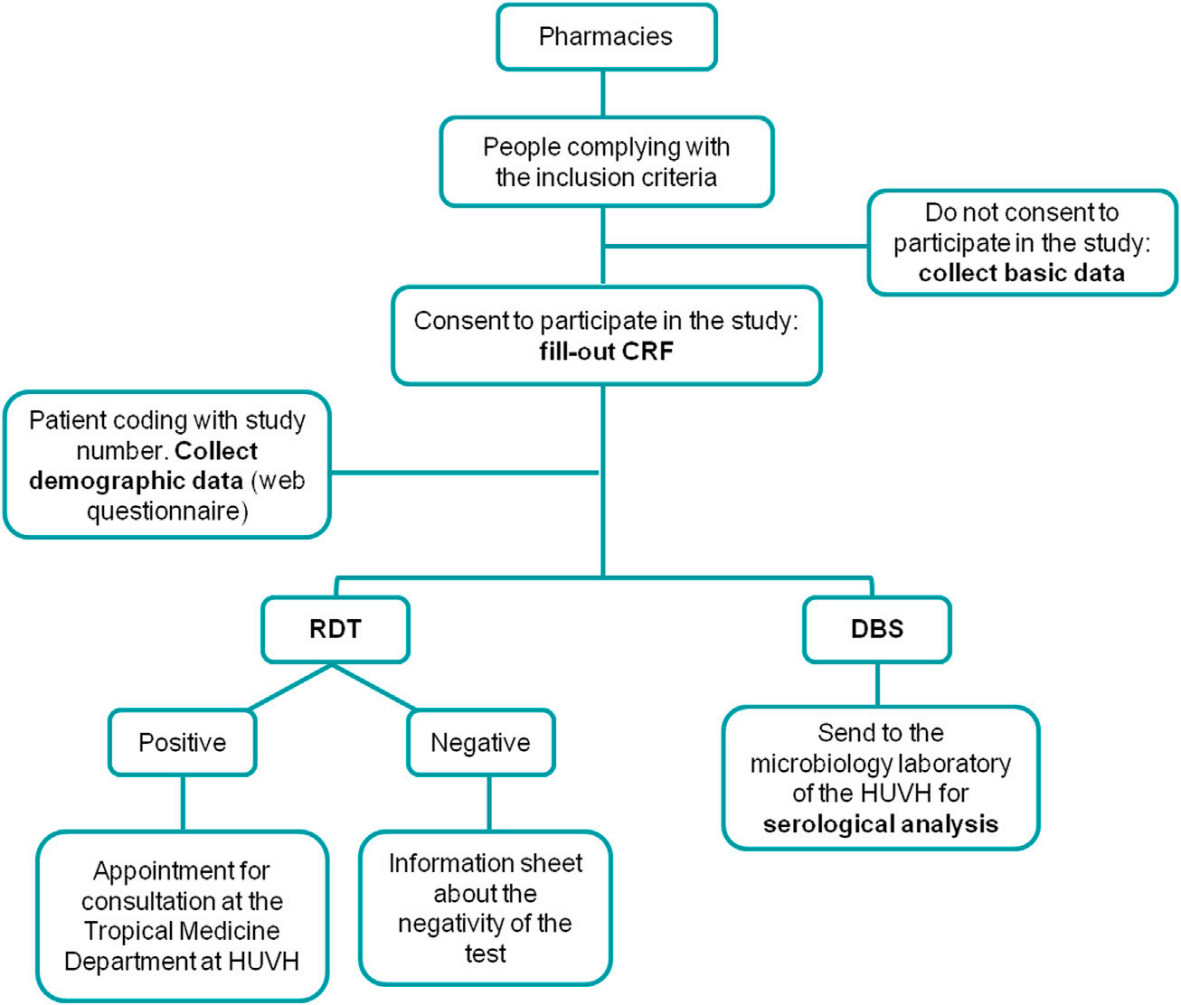
Flow diagram depicting the testing algorithm performed at the pharmacy. (Barcelona, Spain. 2019–2021). CRF, case report form; RDT, Rapid Diagnostic Test; DBS, Dried Blood Spot; HUVH, Vall d’Hebron University Hospital.

When the RDT result was negative, a specific information sheet was given to the participant. Additionally, he/she was informed, using plain language, of the test’s yield, including sensitivity and specificity, and was advised to consult their primary healthcare physician for the corresponding analyses. On the other hand, if the RDT was positive, pharmacists informed the patient about the need for a confirmatory test in a referral hospital. Upon acceptance, the pharmacist could, at that time, schedule an appointment at the HUVH Tropical Medicine Unit for the participant (Figure 2).

#### Laboratory Procedures (RDT and DBS Serology)

A previous analytical study was carried out to determine the sensitivity and specificity of the chosen rapid test, as well as for the analysis of the DBS samples *via* a serological technique (20).

The Trypanosoma Detect™ Rapid Test (InBios International, Inc., Seattle, WA) was selected for the rapid test in community pharmacies and performed according to manufacturer’s instructions. Briefly, one finger-prick blood drop is added to the test strip in the absorbent area, and then three or four drops of buffer solution are added to the same area. The results are read after 10 min.

Regarding DBS samples, between two or three additional drops of blood from the same finger-prick were used to fill one 8-mm-diameter circle on Whatman filter paper card (Whatman 903™ Specimen Collection Paper; GE Healthcare Ltd., Cardiff, United Kingdom). This procedure was repeated to fill a minimum of two circles. If the first finger-prick was not enough to fill the two required circles, the participant was asked about the possibility of making a second finger-prick.

Paper cards were dried at room temperature and then placed in a plastic bag with an anti-humidity sachet. All DBS samples were labeled with a code generated at the time of patient data entry to ensure sample traceability. In accordance with the pharmaceutical distribution system, DBS samples were delivered monthly to the microbiology laboratory of the HUVH for serological analysis.

DBSs were processed for analysis with the Elecsys Chagas assay (Roche Diagnostics, Manheim, Germany) for the determination of anti-*T. cruzi* immunoglobulin G.

#### Community Survey

During March and July 2021, a survey of the target population was carried out to provide information regarding the acceptance of CD screening in CPs. This survey was conducted in the Sants-Montjuïc district, coinciding with the involvement of community agents in the project’s active strategy. Participants were asked whether they would want to be diagnosed for CD in pharmacies and to find out the reasons if not. Oral consent was requested before beginning the survey. No personal data was recorded for this study.

### Ethics

This CD screening program follows the Public Health laws and the Organic Law on Data Protection. Review Board approvals were obtained from the Ethics Committee ofthe Vall d’Hebron Research Institute (PR(AG)230/2018), according to the principles expressed in the Declaration of Helsinki. Informed consent was obtained from all the participants prior to performance of the RDT. Patients were not involved in developing the research question, design or implementation of this analysis.

### Statistical Analysis

Qualitative variables were expressed as absolute frequencies and percentages. Quantitative variables were described as mean or median and standard deviation or interquartile range according to normal distribution. Statistical analyses were done using SPSS Statistics v22.0 (SPSS Inc., Chicago, IL).

## Results

### Pharmacist’s Training

**FIGURE 3:**
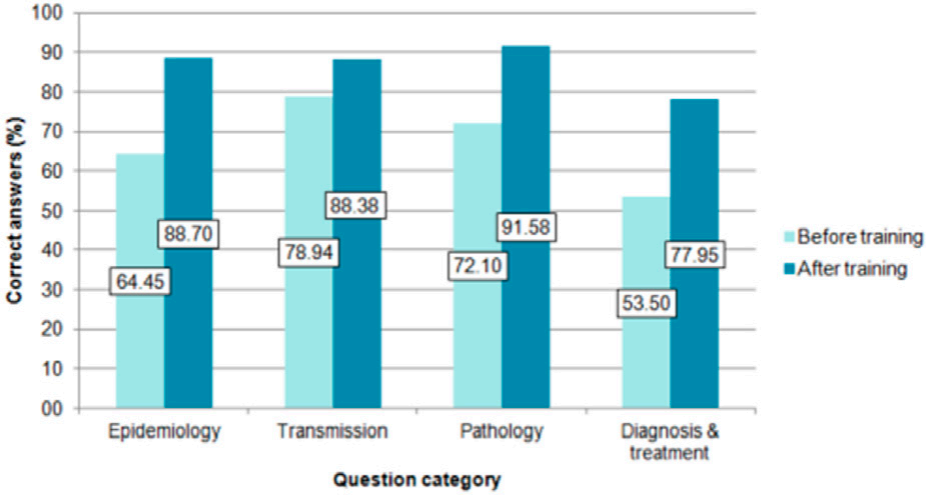
Percentage of correct answers by category before and after the training course. (Barcelona, Spain. 2019–2021). The questions were grouped into category: questions 1 and 2 on epidemiology, questions 3 to 7 on transmission, questions 8 to 12 on pathology, and questions 13 to 18 on diagnosis and treatment.

Thirty-eight pharmacists attended the training course and completed the knowledge survey before and after receiving the training. All question categories showed an increase in the number of correct answers after having completed the course (Figure 3).

The satisfaction survey completed by the participating pharmacists included aspects related to organization, training activity, teaching staff and overall evaluation. The scoring scale ranged from 1 to 4 (4 being very positive). The specific aspects and overall median were between 3 and 4 (data not shown).

### Results From the Pilot Phase and SWOT Assessment

In the pilot phase of the study, a total of 19 pharmacies were included: seven for the passive strategy, six for the active strategy and six for the active-community strategy. A total of 64 patients were included, of which 46 (71.9%) were women, and the median age was 40 years (Inter Quartile Range, 32.75-53). The main countries of origin of the participants were Bolivia (18/64; 28.1%), Ecuador (16/64; 25%) and Peru (12/64; 18.8%). Participants entered the program *via* communication from pharmacy staff (29/64; 45.3%), observation of the material in the pharmacy (27/64; 42.2%) and, to a lesser extent, through an acquaintance or family member (2/64; 3.1%) or information from community agents (1/64; 1.6%). In five cases (7.8%) this information was not registered. The RDT was positive in 2/64 (3.1%) cases. The characteristics of these two cases are resumed in Table 2.

**TABLE 2:** Characteristics of the two patients with a positive RDT result. (Barcelona, Spain. 2019–2021).

	Patient code	
	1	6
Country of origin	Bolivia	Uruguay
Age	43	54
Gender	Female	Male
Serology by DBS	Reactive	Non-Reactive
Conventional serology	Positive	Negative

Of the 64 patients included, a DBS sample was collected in 49 (76.56%) cases. Thirteen samples (13/49; 26.5%) were inadequately collected and could not be processed. Of the remaining 36 DBS samples processed for serological analysis, 26/36 (72.22%) were negative, 1/36 (2.78%) was positive and 9/36 (25%) were classified as invalid, which means that a reading error was obtained in the technique.

Due to the low number of patients recruited, a qualitative study of the acceptance of the project by potential participants was performed in a Latin American population recruited from public places, such as the Bolivian consulate. Of the 94 people surveyed, 27/94 (28.7%) answered that they considered a community pharmacy a suitable facility for CD diagnosis, while 28/94 (29.8%) preferred to be diagnosed in healthcare centers. However, the majority of participants (39/94; 41.5%) answered that they did not want to be tested for CD.

**FIGURE 4:**
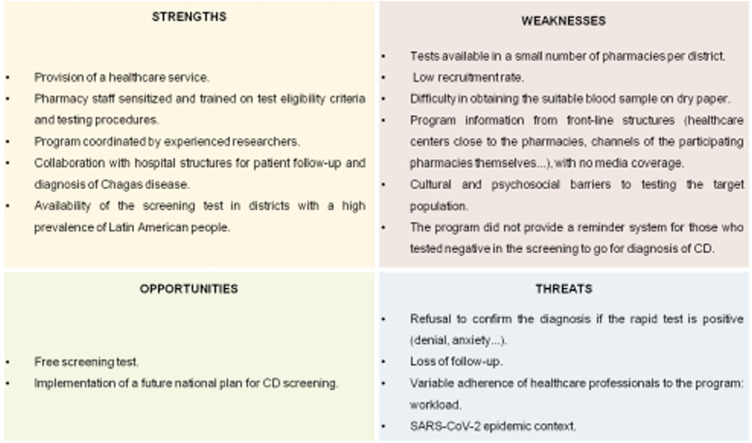
Strengths, weaknesses, opportunities, and threats analysis of the pilot study (Barcelona, Spain. 2019–2021). CD, Chagas disease.

After completion of the pilot experience, a SWOT analysis was applied to improve aspects of the project (Figure 4).

## Discussion

The under-diagnosis of people infected by *T. cruzi* responds to a complex matrix of interrelated factors, mainly attributed to the lack of knowledge of the infection and its transmission, fear and stigma, and a long asymptomatic phase (10). These factors complicate access to a diagnosis and, consequently, to treatment and clinical follow-up care (10). *In situ* screening interventions facilitate access to diagnostic tests and increase accessibility to potentially CD-affected people (21).

In the knowledge survey conducted on pharmacists before the course, the categories of “diagnosis and treatment” and “epidemiology” had the lowest percentage of correct answers. The work of (9), concluded that many physicians outside endemic areas have limited or no knowledge about CD and its implications. It is important to emphasize these aspects since, although the vector is not present in Spain, there are other routes of transmission that are particularly relevant for the transmission of CD in non-endemic areas, especially congenital transmission and *via* organ transplants and blood products (5).

A poor understanding of CD is one of the most important barriers to screening people susceptible to *T. cruzi* infection, especially a lack of accurate information about the disease, including the myth that CD is a synonym for death (22). Moreover, CD diagnosis produces a range of reactions in individuals, from skepticism to fear or anxiety (23). Therefore, a careful approach must be made when offering opportunistic screening to Latin American participants. To this end, our project included the collaboration of a CD “expert patient” with first-hand experience in the disease, who helped design the participant-centered approach and management plan, as well as the communication and dissemination plan (21, 24). Pharmacists were trained in the misconceptions and fears associated with CD. When dealing with the at-risk population, pharmacists are recommended to communicate using clear language, without stereotypes and with scientific rigor. Likewise, they must respect the views and knowledge of the participants. To this end, the figure of the “expert patient,” as well as iconographic materials, were essential for the implementation of this program. The involvement of community agents was intended to reduce the reluctance to participate and increase community engagement with the project. Therefore, their participation was aimed at eliminating the social stigma surrounding CD.

The SWOT analysis of the pilot phase highlighted several strengths of this program, such as coordination by experienced staff and training of pharmacists, as well as the free testing offered. However, several challenges were identified, including the reluctance to know their CD status and the lack of acceptance of positive RDT results.

The study was designed to compare three screening strategies with the hypothesis that pharmacist and community engagement would increase the number of patients screened. After the results from the pilot phase, and according to the participants and pharmacists’ opinions, the passive strategy was dropped.

The COVID-19 pandemic has, in many ways, disrupted the daily lives of people in affected countries, especially regarding social dynamics (25). Countries applied different measures to try to limit the spread of the virus; in Spain, different states of alarm were decreed over time with perimeter closures, quarantines, curfews... as well as the adoption of sanitary measures, such as safety distance, reduced personal contact... Moreover, pharmacies have seen an increase in the services offered as a result of the pandemic, such as antigen and serological tests. This fact may explain, in part, the low recruitment speed observed in this pilot phase of the project.

Rapid diagnostic tests reduce the turnaround time, and with a proper referral procedure, reduce losses to follow-up (26). RDTs are easily implemented at the community level, but the sensitivity reported is not high enough to be used as the sole screening technique. Based on this fact, in the present study, a strategy was selected based on the combination of the RDT with a serology test using DBS, which can reach a sensitivity up to 97% (20).

In 15/64 (23.44%) of the cases it was not possible to collect the DBS sample for serology analysis. This may be due to the fact that, although finger pricking seems to be a simple act, the fact of having to repeat it in order to obtain the DBS sample may be a reason for participants to refuse. In addition, although in 49 cases it was possible to collect DBS samples, 13 of them were collected improperly (either both circles were not full or only one circle was impregnated). Unfortunately, after the pilot phase, the project steering committee decided to remove this procedure as it was considered to hinder the logistics of the screening process.

Similarly, in non-endemic countries, the choice of using RDTs for screening should be followed by confirmation of the results in a reference laboratory (27). Challenges related to the follow-up of patients by specialized units, especially in those with a positive RDT, can be a real problem that can hinder the effectiveness and success of the screening program. In order to try to lose as few patients as possible between screening and subsequent clinical follow-up, the pharmacy booked an appointment for diagnosis in the referral center. Participants not attending the scheduled visit were contacted by phone or e-mail to offer them a new visit.

During the pilot phase, two participants had a positive result on the RDT. Both attended the subsequent visit at the referral unit at HUVH, indicating that acquiring the appointment at the pharmacy is a successful method to avoid the risk of losing the patient. The positive RDT result of patient 6 was not confirmed neither by serology from DBS and serum samples. On the other hand, participants with negative results were carefully explained the meaning of the result and encouraged to perform the diagnosis at his/her primary care center.

Implementation of screening actions for CD, coupled with early management of the disease, is a cost-effective strategy (28); however, cost assessment prior to the large-scale implementation of any strategy is essential for planning and refining procedures and investment efforts (29).

To conclude, DBS sampling and the passive strategy are not suitable for CD screening in community pharmacies. There is a need to expand the number of participating pharmacies and individuals to determine whether conducting a RDT in community pharmacies is an effective screening method to increase access to CD diagnosis in a non-endemic area.

## CHAGAS-FARMACIA Study Group

Francisca Aranzana, Conxita Alsina, Judith Barcelo, Begoña Barenys, Jose M. Bellart, Montserrat Brio, Pere Casanovas, Bibiana de Dou, Eva Falco, Eduard Fernandez-Rajal, Gloria Genis, Enrique Javier Gonzalez, Paula Hernandez, Francesca Iglesias, Antoni Saus, J. Antoni Soriano, Aina Surroca, Marcelo Gabriel Sykuler, Guillermo Vallejo, David Xuriguera.

## Supplementary Material

Supplementary Figure S1 Iconographic material used in this program (in Spanish). **(A)** Poster displayed at the entrance of the pharmacy, visible from the outside. **(B)** Leaflets with brief information about Chagas disease. (Barcelona, Spain. 2019–2021).

Supplementary Figure S2 Guidelines for approaching people from countries endemic for Chagas disease. (Barcelona, Spain. 2019–2021).

## References

[1] World Health Organization. Chagas Disease (American Trypanosomiasis) [Internet] (2020). Available from: http://www.who.int/news-room/fact-sheets/detail/chagas-disease-(american-trypanosomiasis (Accessed May 17, 2022).

[2] Lee BY Bacon KM Bottazzi ME Hotez PJ. Global Economic burden of Chagas Disease: A Computational Simulation Model. Lancet Infect Dis (2013) 13(4):342–8. 10.1016/S1473-3099(13)70002-1 23395248 PMC3763184

[3] Stanaway JD Roth G. The Burden of Chagas Disease: Estimates and Challenges. Glob Heart (2015) 10(3):139–44. 10.1016/j.gheart.2015.06.001 26407508

[4] Navarro M Reguero L Subirà C Blázquez-Pérez A Requena-Méndez A. Estimating Chagas Disease Prevalence and Number of Underdiagnosed, and Undertreated Individuals in Spain. Trav Med Infect Dis (2022) 47:102284. 10.1016/j.tmaid.2022.102284 35245657

[5] Pérez-Molina J Molina I. Chagas Disease. Lancet (2018) 391:82–94. 10.1016/S0140-6736(17)31612-4 28673423

[6] Conselleria de Salut CV. Enfermedad de Chagas importada. Protocolo de actuación en la Comunitat Valenciana. Valencia: Enfermedades Emergentes (2009).

[7] Galicia X. Protocolo de cribado da enfermidade de chagas en mulleres embarazadas. Santiago de Compostela: Xunta de Galicia (2014).

[8] Ciruela Navas P Jané Checa M. Protocolo De Cribado, Diagnóstico Y Tratamiento De La Enfermedad De Chagas En Mujeres Embarazadas Latinoamericanas Y En Sus Hijos. Agència Salut Pública Catalunya [Internet] (2018). Available from: http://canalsalut.gencat.cat/web/.content/_A-Z/C/chagas/documents/arxius/protcolcribratgeidiagnostic_cast.pdf (Accessed May 17, 2022).

[9] Iglesias-Rus L Romay-Barja M Boquete T Benito A Blasco-Hernández T. The Role of the First Level of Health Care in the Approach to Chagas Disease in a Non-Endemic Country. Plos Negl Trop Dis (2019) 13(12):e0007937–16. 10.1371/journal.pntd.0007937 31841503 PMC6913928

[10] Gómez i Prat J Peremiquel-Trillas P Claveria Guiu I Choque E Oliveira Souto I Serre Delcor N A Community-Based Intervention for the Detection of Chagas Disease in Barcelona, Spain. J Community Health (2019) 44(4):704–11. 10.1007/s10900-019-00684-z 31222620

[11] Pérez-Molina JA Norman F López-Vélez R. Chagas Disease in Non-Endemic Countries: Epidemiology, Clinical Presentation and Treatment. Curr Infect Dis Rep (2012) 14(3):263–74. 10.1007/s11908-012-0259-3 22477037

[12] World Health Organization. Guidelines for the Diagnosis and Treatment of Chagas Disease. Washington, DC: PAHO (2019).

[13] Shah V Ferrufino L Gilman RH Ramirez M Saenza E Malaga E Field Evaluation of the InBios Chagas Detect Plus Rapid Test in Serum and Whole-Blood Specimens in Bolivia. Clin Vaccin Immunol (2014) 21(12):1645–9. 10.1128/CVI.00609-14 PMC4248774 25274804

[14] Angheben A Buonfrate D Cruciani M Jackson Y Alonso-Padilla J Gascon J Rapid Immunochromatographic Tests for the Diagnosis of Chronic Chagas Disease in At-Risk Populations: A Systematic Review and Meta-Analysis. Plos Negl Trop Dis (2019) 13(5):e0007271–15. 10.1371/journal.pntd.0007271 31150377 PMC6561601

[15] McClendon-Weary B Putnick DL Robinson S Yeung E. Little to Give, Much to Gain—What Can You Do with a Dried Blood Spot? Curr Environ Health Rep (2020) 7(3):211–21. 10.1007/s40572-020-00289-y 32851603 PMC7500853

[16] Ayorinde AA Porteous T Sharma P. Screening for Major Diseases in Community Pharmacies: A Systematic Review. Int J Pharm Pract (2013) 21(6):349–61. 10.1111/ijpp.12041 23683090

[17] Fernandez-Lopez L Reyes-Urueña J Conway A Saz J Morales A Quezadas J The Contribution of HIV point-of-care Tests in Early HIV Diagnosis: Community-Based HIV Testing Monitoring in Catalonia, 1995 to 2018. Eurosurveillance (2020) 25(43):1–11. 10.2807/1560-7917.ES.2020.25.43.1900424 PMC7596919 33124552

[18] Ibañez-Sanz G Milà N Vidal C Rocamora J Moreno V Sanz-Pamplona R Positive Impact of a Faecal-Based Screening Programme on Colorectal Cancer Mortality Risk. Plos One (2021) 16(6):e0253369–14. 10.1371/journal.pone.0253369 34191813 PMC8244848

[19] Institut d’Estadística de C. Población Extrangera [Internet] (2020). Available from: https://www.idescat.cat/poblacioestrangera/?b=10&geo=mun%3A080193&nac=d314#Plegable=geo (Accessed May 17, 2022).

[20] Silgado A Gual-Gonzalez L Sánchez-Montalvá A Oliveira-Souto I Goterris L Serre-Delcor N Analytical Evaluation of Dried Blood Spot and Rapid Diagnostic Test as a New Strategy for Serological Community Screening for Chronic Chagas Disease. Front Cell Infect Microbiol (2021) 11:736630. 10.3389/fcimb.2021.736630 34604116 PMC8479190

[21] Gómez i Prat J Peremiquel-Trillas P Guiu IC Mendivelso JC Choque E de los Santos JJ Comparative Evaluation of Community Interventions for the Immigrant Population of Latin American Origin at Risk for Chagas Disease in the City of Barcelona. Plos One (2020) 15(7):e0235466–15. 10.1371/journal.pone.0235466 32663211 PMC7360029

[22] Avaria A Gómez i Prat J. Si Tengo Chagas es Mejor que me Muera El Desafío de Incorporar una Aproximación Sociocultural a la Atención de Personas Afectadas por la Enfermedad de Chagas. Enferm Emerg (2008) 10(1):40–5.

[23] Echeverría LE Marcus R Novick G Sosa-Estani S Ralston K Zaidel EJ WHF IASC Roadmap on Chagas Disease. Glob Heart (2020) 15(1):26–31. 10.5334/gh.484 32489799 PMC7218776

[24] Velarde-Rodriguez M Avaria-saavedra A Gomez-i-Prat J Jackson Y De Oliveira Junior AW Camps-carmona B Need of Comprehensive Health Care for *T. Cruzi* Infected Immigrants in Europe. Rev Soc Bras Med Trop (2009) 42(2):92–5.

[25] Fontana Sierra L. Pandemic and Rearticulation of Social Relations. Periferia (2020) 25(2):101. 10.5565/rev/periferia.770

[26] Gubbins PO Klepser ME Dering-Anderson AM Bauer KA Darin KM Klepser S Point-of-care Testing for Infectious Diseases: Opportunities, Barriers, and Considerations in Community Pharmacy. J Am Pharm Assoc (2014) 54(2):163–71. 10.1331/JAPhA.2014.13167 24632931

[27] Sánchez-Camargo CL Albajar-Viñas P Wilkins PP Nieto J Leiby DA Paris L Comparative Evaluation of 11 Commercialized Rapid Diagnostic Tests for Detecting *Trypanosoma Cruzi* Antibodies in Serum banks in Areas of Endemicity and Nonendemicity. J Clin Microbiol (2014) 52(7):2506–12. 10.1128/JCM.00144-14 24808239 PMC4097696

[28] Requena-Méndez A Bussion S Aldasoro E Jackson Y Angheben A Moore D Cost-effectiveness of Chagas Disease Screening in Latin American Migrants at Primary Health-Care Centres in Europe: a Markov Model Analysis. Lancet Glob Health (2017) 5(4):e439–47. 10.1016/S2214-109X(17)30073-6 28256340

[29] Hoomans T Severens JL. Economic Evaluation of Implementation Strategies in Health Care. Implement Sci (2014) 9:168. 10.1186/s13012-014-0168-y 25518730 PMC4279808

