# Supplementary figures and images for: Opportunistic Community Screening of Chronic Chagas Disease Using a Rapid Diagnosis Test in Pharmacies in Barcelona (Catalonia, Spain): Study Protocol and Pilot Phase Results
Source: Int J Public Health. 2022 Nov 30;67:1605386. doi: 10.3389/ijph.2022.1605386 (PMC9747760; doi:10.3389/ijph.2022.1605386)

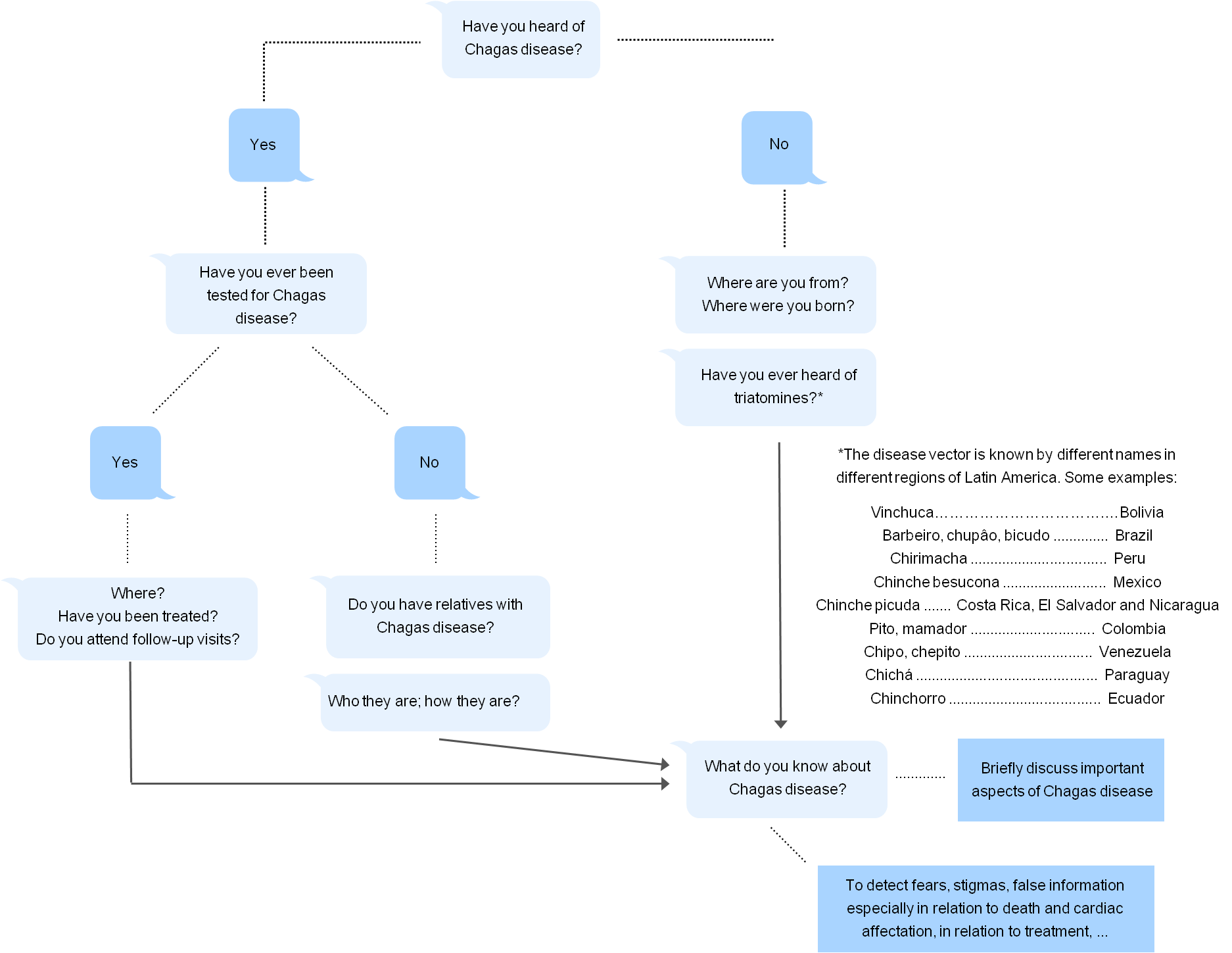

Supplement: Supplementary file 1 [file Image2.TIF]

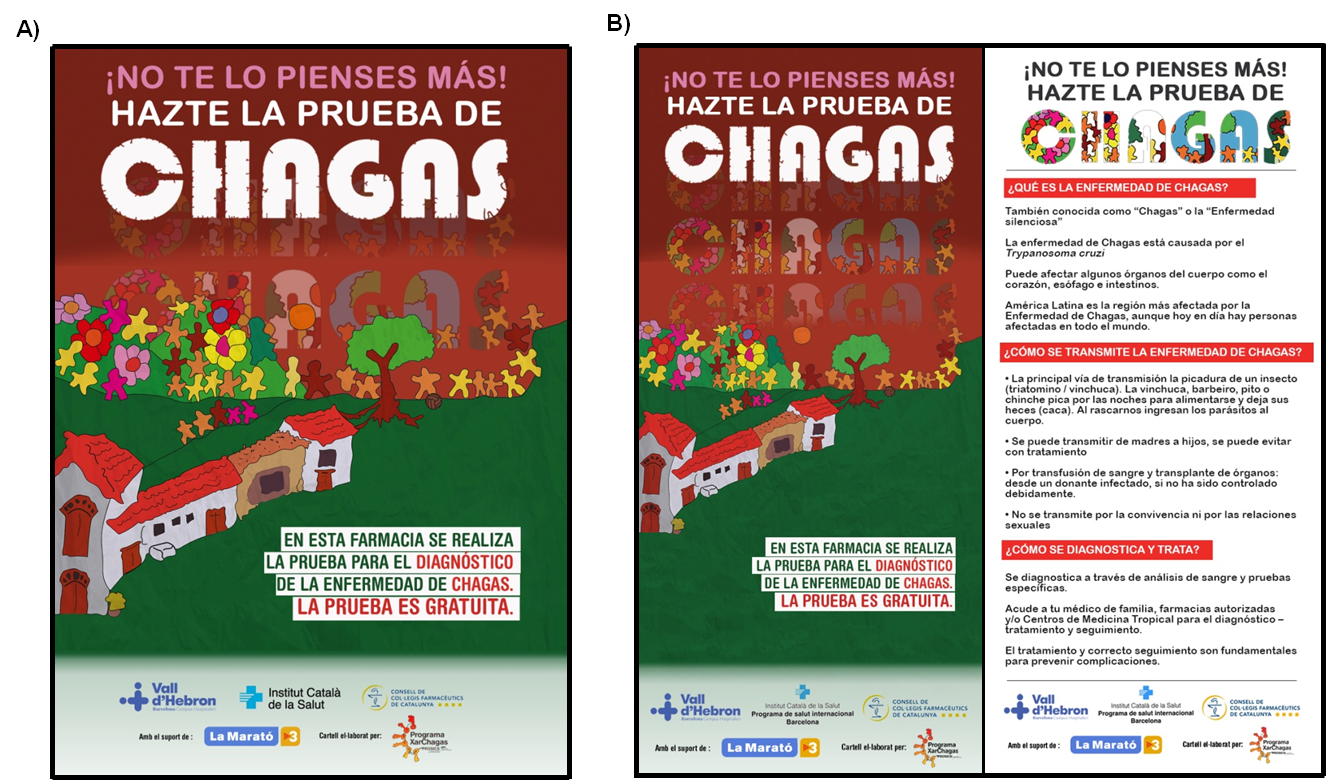

Supplement: Supplementary file 2 [file Image1.TIF]
